# Spatial Distribution of Heavy Metals and the Environmental Quality of Soil in the Northern Plateau of Spain by Geostatistical Methods

**DOI:** 10.3390/ijerph14060568

**Published:** 2017-05-26

**Authors:** Fernando Santos-Francés, Antonio Martínez-Graña, Carmelo Ávila Zarza, Antonio García Sánchez, Pilar Alonso Rojo

**Affiliations:** 1 Department of Soil Sciences, Faculty of Agricultural & Environmental Sciences, University of Salamanca, Avenue Filiberto Villalobos, 119, 37007 Salamanca, Spain; fsantos@usal.es (F.S.-F.); palrojo@usal.es (P.A.R.); 2 Department of Geology, Faculty of Sciences, University of Salamanca, Plaza de la Merced s/n., 37008 Salamanca, Spain; 3 Department of Statistics, Faculty of Agricultural & Environmental Sciences, University of Salamanca, Avenue Filiberto Villalobos, 119, 37007 Salamanca, Spain; caaz@usal.es; 4 Department of Environmental Geochemistry, Institute of Natural Resources and Agrobiology—IRNASA (C.S.I.C.), Calle Cordel de Merinas 40, 37008 Salamanca, Spain; antonio.gsanchez@irnasa.csic.es

**Keywords:** soil, heavy metals, spatial distribution, environmental quality, Kriging, principal component analysis, Northern Plateau of Spain

## Abstract

The environmental quality of soil in the central part of the Northern Plateau of Spain has been analyzed by studying the heavy metal content of 166 samples belonging to the horizons A, B and C of 89 soil profiles. The analysis to assess the environmental risk of heavy metals in the soil was carried out by means of the spatial distribution of nine heavy metals and the use of several pollution indices. The results showed that the concentration values of heavy metals ($\bar{x}$ ± S) in the superficial soil horizons were the following: With a total of 6.71 ± 3.51 mg·kg^−1^, the contents of Cd is 0.08 ± 0.06 mg·kg^−1^, Co is 6.49 ± 3.21 mg·kg^−1^, Cu is 17.19 ± 10.69 mg·kg^−1^, Cr is 18.68 ± 12.28 mg·kg^−1^, Hg is 0.083 ± 0.063 mg·kg^−1^, Ni is 12.05 ± 6.76 mg·kg^−1^, Pb is 14.10 ± 11.32 mg·kg^−1^ and Zn is 35.31 ± 14.63 mg·kg^−1^. These nine metals exceed the values of the natural geological background level of Tertiary period sediments and rocks that form part of the Northern Plateau in Spain. Nemerow and Potential Ecological Risk indices were calculated, with the “improved” Nemerow index allowing pollution within the soil superficial horizons to be determined. The data obtained indicated that the majority of the soil (54.61%) showed low to moderate contamination, 22.31% showed moderate contamination and 21.54% of the samples were not contaminated. If we consider the Potential of Ecological Risk Index (RI), the largest percentage of soil samples showed low (70.79%) to moderate (25.38%) ecological risk of potential contamination, where the rest of the soil presented a considerable risk of contamination. The nine trace elements were divided into three principal components: PC_1_ (Cu, Cr, Ni, Co and Zn), PC_2_ (As and Hg) and PC_3_ (Cd). All metals accumulated in the soil came from parent rock, agricultural practices and the run-off of residual waters towards rivers and streams caused by industrial development and an increase in population density. Finally, cartography of the spatial distribution of the heavy metal contents in the soil of the Northern Plateau of Spain was generated using Kriging interpolation methods. Furthermore, the total heavy metal contents in three soil orders present in the area, namely Entisols, Inceptisols, and Alfisols, were analyzed. Other soil parameters, such as the organic matter content, pH, clay content and cation exchange capacity, was measured to determine their influence on and correlation with the heavy metal contents.

## 1. Introduction

Toxic heavy metals present in soil, such as Cd, Cu, Pb, Zn, Ni, Cr, and As, potentially affect the environment, because they are durable and non-biodegradable. The accumulation of toxic metals has an impact on natural ecosystems and can present a threat to humans through the food chain, owing to their bioaccumulation in food [1,2,3,4]. 

The natural concentrations of trace elements in soil play a key role in controlling the impact caused by humans and in the level of soil quality of an area [5,6,7]. Studies of soil contaminated by heavy metals have identified areas with a high risk of pollution through the analysis of atypical local spatial values [8,9]. The criteria and methods are diverse. However, spatial distribution analysis, localization of the sources of contamination and the assessment of soil quality are of particular interest [10,11]. The accumulation of heavy metals in soil is generally influenced by many environmental variables, including the parent rock and properties of the soil [12,13,14], as well as human activities, such as industrial production, traffic and agriculture. Extensive areas of land can be polluted by heavy metals released from waste incineration, industrial residual waters in addition to the application of fertilizers, pesticides and sewage sludge that contain heavy metals [15,16,17]. When different sources of contamination are present and their products are dispersed, the spatial variability of the heavy metal concentrations allows possible sources of contamination to be identified and the remediation strategies to be proposed. Independent of origin, the accumulation of heavy metals can damage soil quality and the efficiency of agricultural products, which can have to a negative impact on human health and ecosystems [18].

The assessment of environmental quality, based on the heavy metal contents in soil, involves the use of different pollution indices: the geoaccumulation index, the pollution load index, the potential of ecological risk index, the Nemerow index (Supplementary Materials) and so on. The geostatistical multivariate analysis implemented using geographic information systems (GIS) allows for more meaningful and accurate information. In soil pollution studies, the economic cost in addition to sampling and soil analysis should be taken into account, since dense and repetitive sampling is usually not very practical. With the aim to avoid these types of problems, the creation of maps representing the spatial distribution of contamination uses methods of spatial interpolation. The geostatistical method “Kriging” is currently used successfully to describe and predict the spatial variability of certain soil parameters and to predict values of non-sampled areas [19,20,21,22,23,24,25,26,27,28]. With the traditional statistical method, it is necessary for the data to be subjected to a series of assumptions: independent observations, wide and repeated sampling, etc. 

Over the last decades, the research on the environmental geochemistry of heavy metals, owing to their toxicity and durability in the environment, has greatly advanced [29,30,31]. Recently, the analysis of principal components has been used in many fields to study soil, including pollution due to heavy metals. This is because this type of analysis shows the existing relationships between heavy metals in different spatial dimensions and determines the relative importance of each metal [32,33,34,35]. 

The presence of values that exceed the background threshold level of a given geological area is the traditional method used to characterize the degree of soil pollution [36]. High concentration levels of some heavy metals may occur through natural contamination (minerology and the chemical composition of the parent rock) or through anthropic pollution (agricultural land use, discharge of residual waters originating from industrial and urban areas towards rivers and streams, etc.).

The aim of the present work was to: quantify the environmental quality of the soil of the Northern Plateau of Spain by analyzing its heavy metal contents; assess the environmental risk of heavy metals by calculating various pollution indices and by analyzing their spatial and vertical distribution (within deep layers and throughout the entire soil profile); determine the correlation between the heavy metal contents and some soil parameters, such as the organic matter content, pH, clay content and cation exchange capacity; and calculate the average content of heavy metals, according to the type of parent rock and land use.

## 2. Materials and Methods 

### 2.1. Study Area

The Northern Plateau, an area that forms part of the Spanish Central Plateau, is located to the north of the Central System and includes most of the autonomous regions of Castile and Leon. The study area, located within the Northern Plateau, occupies approximately 25,050 km^2^, which includes the whole area of the province of Valladolid and most of Palencia and Burgos. This area has a Mediterranean climate with cold and rainy winters in addition to hot and dry summers. The average annual rainfall is between 400 and 550 mm, while the average annual temperature is between 11 °C and 12.5 °C. The annual precipitation is less than the amount of potential evapotranspiration, which is between 660 and 730 mm, and occurs mainly between November and February. 

**Figure 1:**
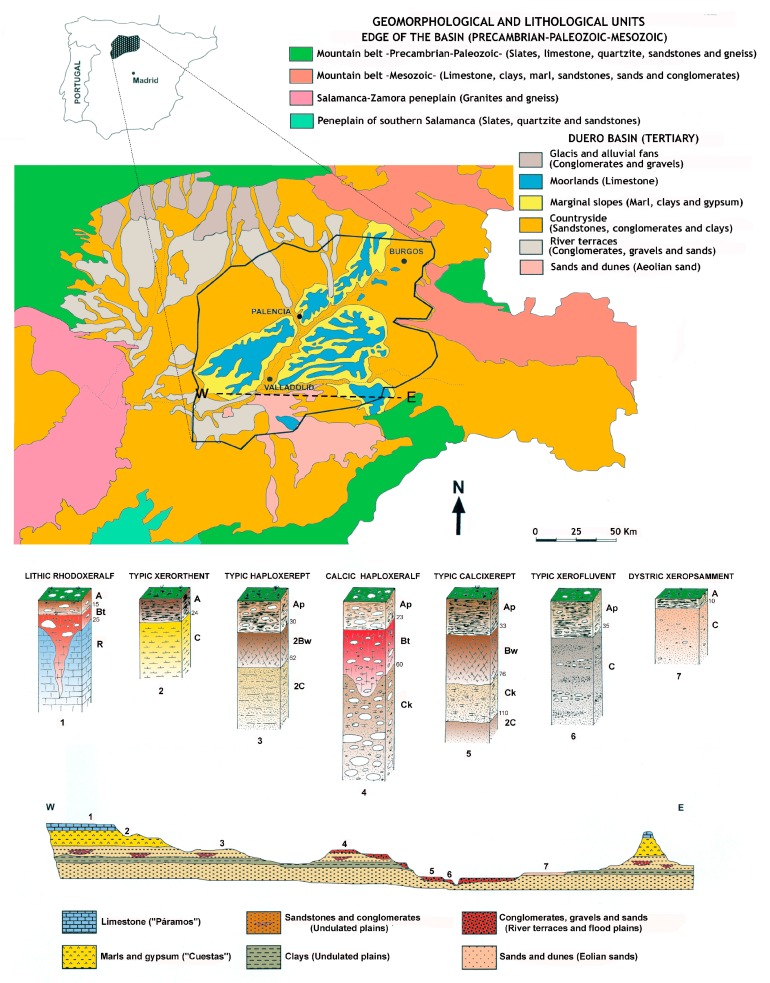
Geomorphological and lithological units of the Northern Plateau (Spain), location of study area (**top**) as well as the scheme that relates lithology, geomorphology and soil (**bottom**).

From a geological view point, this area belongs to the great Cenozoic depression of the Duero Basin, caused by the collapse of the Hercynian base and filled up throughout the entire Tertiary period with sediments originating from the continent with a tabular–horizontal arrangement. This basin is surrounded by mountains (Figure 1) [37].

In each lithological unit, an inventory of the most representative types of soil was conducted, with the profiles described and classified, according to the U.S. soil taxonomy [38,39]. The geographical distribution of the soil originating from the Duero basin is greatly influenced by the following shape-forming factors: lithology, relief and period or age of the geomorphological surfaces (Figure 1).

(a) Calcareous wastelands: this unit is comprised of the Late Miocene limestones that form a flat surface or a calcareous platform characteristic of wastelands, depending on their specific horizon. The physiographical features show a predominance of little-developed and dense soil (Xerorthents) as well as moderately thick soil (Rhodoxeralf and Haploxeralf).

(b) Gypsiferous loam slopes: a unit comprised mainly of soft materials (white and grey loams as well as small amounts of clays and gypsum) from the Late Miocene period. The soil comprising these hills and slopes has a clay-like texture, are highly chalky, and exhibit different degrees of erosion. These are related directly to the gradient of the slope, ranging from Haploxerepts to Xerorthents.

(c) Countryside or undulating plains: a unit shaped by detrital material and comprised of alternating sandstones and silty clays. This is slightly chalky and from the Miocene period (the so-called *Tierra de Campos* facies). The most characteristic soil on top of this type of lithology has been classified as Haploxerepts.

(d) Fluvial terraces: these terraces represent the remains of ancient riverbeds (Pleistocene), tiered to different heights. These run parallel and in the longitudinal direction along the course of the most abundant rivers (Duero, Pisuerga, Cea, and so on) and are comprised of generally well-cemented conglomerate deposits of quartzite, quartz and limestone in most of the ancient riverbeds. The lower or more modern riverbeds are comprised of gravel with sandy intercalations. The soil situated above this type of lithology has been classified as Palexeralfs and Haploxeralfs [40]. 

(e) Flood plains or water meadows of large rivers: these sediments are generally made up of silts, sand and loose gravel. The most characteristic soil of this unit has been classified as Calcixerepts and Xerofluvents.

(f) Wind sands: throughout the entire Quaternary period (Pleistocene-Holocene), wind gave rise to various erosional and sedimentation processes, creating loose arkose deposits with round grains. In some cases, these form dunes 15 m in depth, although these do not reach even one meter in other places. The sandy areas are usually alluvial deposits from rivers and sands from the Utrillas facies (Cretaceous), belonging to the Mesozoic period of the Iberian mountain range. This area is covered by white loose sands, forming soil classified as Xeropsamments.

### 2.2. Soil Samples

The soil sampling sites were chosen, based on the different lithological units. In each site, individual samples were randomly taken for each of the horizons (A, B and C). In each horizon, five were collected and mixed to give homogeneity, obtaining a total of 1 kg of soil. This was subsequently dried at room temperature and then passed through a 2 mm sieve. In each site, individual samples are collected for each of the horizons (A, B and C). In each horizon, five sub-samples are collected and mixed to give homogeneity, obtaining a total sample of 1 kg. The number of samples was 166 in total, which belongs to the soil profiles from the horizons A, B and C. Considering the five sub-samples of each horizons, the total number of collected samples is 830. The physical and chemical properties were determined using the traditional soil analysis methods [41]: organic matter by oxidation with potassium dichromate, granulometric analysis using the Robinson pipette method, pH (water 1:1) and cation exchange capacity -CEC- using the ammonium acetate method (Table 1). 

The total contents of heavy metals in soil have been analyzed according to the procedure recommended by the European Union ISO standard 11466. Extraction was performed with a mixture of nitric acid and hydrochloric acid in a microwave oven, with determination by ICP-MS model Elan 6000 of Perkin–Elmer. The analysis was carried out by the Chemical Analysis Service of the University of Salamanca through the digestion of the samples in a microwave oven (Millestone mod. Ethos Plus Microwave Lastation), using the standard method, USEPA method 3052. For the calibration of the equipment, standard solutions (panreac) of 1000 mg/L of all the metals analyzed were used, which were calibrated from 10 to 100 ppb. The data of the relative standard deviation (RSD) have all been less than 3% for all elements analyzed.

In addition, to check the quality of the analysis, blind duplicates were analyzed. The quality control of the data showing detection limits, accuracy and precision. For the elements: As, Cd, Co, Cu, Cr, Hg, Ni, Pb and Zn, the detection limit are: 0.1, 0.01, 0.1, 0.01, 0.5, 5, 0.1, 0.01, and 0.1 respectively, the accuracy is 2.3%, 0.6%, 1.4%, 3.2%, 7.6%, 1.3%, 1.7%, 1.4% and 2.8% respectively and the precision is 0.5%, 0.2%, 0.4%, 0.7%, 1.9%, 0.3%, 0.4%, 0.5% and 0.8% respectively. Nearly 6% of the samples were analyzed in duplicates internal and external controls. The accuracy error and the precision error were estimated as 2.5% and 0.6% respectively, which denote high confidence in the results.

### 2.3. Statistical Analysis Samples

The statistical analysis was carried out using the SPSS v.23.0 software (Armonk, NY, USA). A descriptive analysis was obtained using the following variables: arithmetic mean, geometric mean, median, range, standard deviation, variable coefficient and kurtosis. In addition, several pollution indices were calculated to determine the level of contamination of heavy metals in the soil. Finally, a multivariate analysis was conducted using the principal components method to identify relationships between the heavy metal concentrations in the soil and to obtain information concerning the possible sources responsible for the presence of the heavy metals [42]. 

The GIS analysis (ArcGis v10.4), with the extensions of spatial analysis tools and geostatistical analysts, calculated the degree of spatial variability of each of the heavy metals and the pollution indices (the “improved” Nemerow Pollution Index and the Potential of Ecologic Risk Index), using standard Kriging to determine interpolation and the subsequent semivariogram analysis [43]. Kriging is an advanced geostatistical method that generates a surface area, based on a set of dispersed values. It can also generate an interactive study of the spatial characteristics, which is soil pollution caused by the presence of heavy metals in this case. In contrast to the deterministic interpolation methods of IDW and Spline, which directly depend on the surrounding measured values that determine the size of the resulting surface area, the geostatistical method Kriging is based on statistical models that include autocorrection. In essence, this is the statistical relationship between the points measured. The primary difference between Kriging and IDW (Inverse Distance Weight) is that IDW is the interpolation method that only takes distance dependency between soil samples into account. In comparison, Kriging is the most enhanced interpolation method that takes not only distance dependency, but also directional dependency into account. In the assessment of contaminated soil, this statistical geographic method generates a predicted surface area of the distribution of the heavy metals with certainty and predictive accuracy. A map of the predicted surface area was built using an equation (Equation (1)) that reveals the rules of dependency between points and then predicts the spatial or geographic distribution.

(1) $$Z\;\left(S_{0}\right)=\overset{N}{\underset{i=1}{\sum }}\lambda_{i}Z\;\left(S_{i}\right)$$

where *Z (s_i_)* is the value measured in location *i*; *λ_i_* is an unknown weighting of the measured value in location *i*; *s*_0_ is the location of the prediction; and *N* is the number of measured values.

The two steps of the process carried out in this work are as follows: to create the variograms and the covariance functions to calculate the values dependent on statistics (spatial autocorrection) that are dependent on the model of autocorrection (adequacy of fit) and to determine unknown values (prediction). In this work, the Kriging method applied was standard, which presumes that the distance or the direction between sampling sites reflect a spatial correlation that can be used to explain variations within the surface area. The semivariogram model uses the spherical mathematical model, which adjusts the mathematical function to 12 sitepoints in a search radius variable [44].

## 3. Results

### 3.1. Concentration of Heavy Metals in Soil

The mean values of the heavy metals obtained as a result of the statistical analysis of the soil of the Northern Plateau of Spain can be seen in Table 2. These average values are similar to those described for other countries [45,46].

The mean values of As, Cd, Ni, Cr, Pb, Cu, Hg, Co and Zn in the soil were 1.18, 1.19, 1.40, 1.49, 1.50, 1.54, 1.55 and 1.67 times higher, respectively, than their natural geological background levels. All these data show that there is an evident tendency towards the accumulation of heavy metals in the soil of the region studied.

The variation coefficient of Pb and Hg, which were 0.80 and 0.76 respectively, were the highest, implying that these two metals had the greater variability throughout the area studied compared to the other metals also present in the soil: As, Cd, Co, Cu, Cr, Ni, Pb and Zn (0.52, 0.78, 0.49, 0.62, 0.66, 0.76, 0.56 and 0.41), which also showed high values (>41%) dispersed along the sampled areas.

### 3.2. Assessment of the Environmental Risks: Contamination

The Pollution factor (*P_i_*) quantifies the pollution of one individual metal, *P_i_* = *C_i_*/*B_i_*, where “*C_i_*” is the concentration of the measured contaminant and “*B_i_*” is the geological background level, which allows the levels of the different metals to be determined.

The Nemerow index (*I_N_*) assesses soil quality, according to the degree of pollution of various metals and taking into account the pollution factor. This was defined by the following equation (Equation (2)):

(2) $$I_{N}=\sqrt{\left({P_{i_{max}}}^{2}+{P_{i_{ave}}}^{2}\right)}$$

where “*Pi_max_*” is the maximum value and “*Pi_ave_*” is the mean value of the pollution factors of all the metals (Table 3).

According to the results of this pollution factor, four categories were established: 34% of the samples studied have low pollution (*Pi* < 1), 59.15% have moderate pollution (1 ≤ *Pi* < 3), 5.65% have high pollution (3 ≤ *Pi* < 6) and 4.62% have very high pollution (*Pi* > 6). This data show that a large percentage of the soil was polluted to a low to moderate degree and that only a small amount of soil was highly polluted. 

The levels of pollution according to the Nemerow index in the study area show that the majority of the soil presented low to moderate contamination, while only a small percentage was non-contaminated or slightly contaminated. The soil considered as “contaminated” (Class III, 1 ≤ *I_N_* < 2) represented 46.15% of the samples analyzed, the soil “moderately contaminated” (Class IV, 2 ≤ *I_N_* < 3) represented 36.92% and the “highly contaminated” soil (Class V, *I_N_* > 3) represented 14.62%. The slightly contaminated soil (Class II, 0.7 ≤ *I_N_* < 1) were less represented and only made up 0.77% of the samples. In comparison, non-contaminated or clean soil (Class I, *I_N_* < 0.7) made up 1.54% of the samples analyzed.

The geo-accumulation index (Igeo) is calculated using the following equation (Equation (3)):

(3) Igeo=log2(Ci1.5Bi)

where “*C_i_*” is the concentration measured of metal “*i*” examined in the soil and “B” is the geological background level of metal “*i*”. The factor 1.5 was used to correct possible variations in the background values of the specific metal in the environment. The resulting values were then classified as non-contaminated (Igeo ≤ 0, Class 0), from non-contaminated to moderately contaminated (0 < Igeo ≤ 1, Class 1), moderately contaminated (1 < Igeo ≤ 2, Class 2), from moderately contaminated to highly contaminated (2 < Igeo ≤ 3, Class 3), highly contaminated (3 < Igeo ≤ 4, Class 4), from highly contaminated to extremely contaminated (4 < Igeo ≤ 5, Class 5) and extremely contaminated (Igeo > 5, Class 6).

The geo-accumulation indices (Igeo) of the heavy metals studied allowed the pollution index, with only one factor to be analyzed in order to assess the presence of each specific metal and the corresponding level of contamination in the area studied. Furthermore, the “improved”, multifactorial Nemerow index (*I_MN_*) showed the overall degree of pollution caused by the simultaneous presence of the nine heavy metals (Table 4).

After considering the geo-accumulation index, it was observed that the levels of heavy metal contamination of the soil in the superficial horizons of the Northern Plateau did not include Classes 4, 5 and 6 (from strongly polluted to extremely polluted). The majority of the soil contents were situated within Classes 0 (56.23% of samples), 1 (36.07%), 2 (7.79%) and 3 (2.34%) (unpolluted to moderately polluted). 

The “improved” Nemerow index (*I_MN_*) substitutes the contamination factor (*P_i_* = *C_i_*/*B_i_*) for the equation Igeo = log_2_ (*C_i_*/1.5 *B_i_*), in which the *I_MN_* is calculated using the following equation (Equation (4)):

(4) IMN=12(Igeomax2+Igeoave2)

where “Igeomax” is the maximum value of the Igeo of all metals in a sample and “Igeoave” is the arithmetic mean of the Igeo. After taking the Nemerow index into account, the soil contents within the study area ranged from contaminated to highly contaminated. However, using the “improved” Nemerow index (*I_MN_*), the soil was shown to be moderately to heavily contaminated. Specifically, 21.54% of the soil was uncontaminated (*I_MN_* < 0.5, Class 0), 54.61% was between uncontaminated to moderately contaminated (0.5 ≤ *I_MN_* < 1, Class 1), 22.31% was moderately contaminated (1 ≤ *I_MN_* < 2, Class 2) and 1.54% was moderately to heavily contaminated (2 ≤ *I_MN_* < 3, Class 3). Furthermore, there are no samples belonging to Class 4, which is heavily contaminated (3 ≤ *I_MN_* < 4); Class 5, which is heavily to extremely contaminated (4 ≤ *I_MN_* < 5); and Class 6, which is extremely contaminated (*I_MN_* > 5).

The Potential of Ecological Risk Index (*Er*) assesses the toxicity of some trace elements in sediments [47] and is currently widely applied in the analysis of soil [48,49,50,51]. Soil contaminated by heavy metals can cause serious risk to the environment and to human health owing to diverse interactions (agriculture, cattle breeding, etc.), which ultimately allow highly toxic heavy metals to enter into the food chain. The excessive accumulation of heavy metals in soil used for agricultural purposes can affect the quality and safety of food. Furthermore, this can increase the risk of serious diseases (cancer, kidney and liver damage, etc.) as well as causing environmental impact by combining environmental chemistry with biological and ecological toxicology [52].

To calculate the Potential of Ecological Risk index (*Er*) for each metal, the following equation was used (Equation (5)):

(5) $$Er_{m}=Tr_{m}\cdot P_{i_{m}}$$

where “*Tr*” is the toxicity coefficient of each metal, which standard values are: Hg = 40, Cd = 30, As = 10, Co = 5, Cu = 5, Ni = 5, Pb = 5, Cr = 2 and Zn = 1. 

The toxicity response index of all of the heavy metals studied was calculated using the following equation (Equation (6)):

(6) $$RI=\overset{n}{\underset{i=1}{\sum }}Er$$

The Potential of Ecological Risk (*RI*) reflects the general situation of the contamination caused by the simultaneous presence of the nine heavy metals (Table 5).

The categories established for the RI index for classifying risk are: low ecological risk of potential contamination (*RI* < 150), moderate ecological risk 150 ≤ *RI* < 300), considerable ecological risk (300 ≤ *RI* < 600) and very high ecological risk (*RI* > 600). It was observed in the area studied that a large percentage of the soil samples (96.92%) were within low to moderate ecological risk of potential contamination, with only a small proportion (3.08%) posing a considerable risk of potential contamination.

### 3.3. Analysis of Principal Components

Using a data matrix comprised of the concentrations obtained for each heavy metal in each sample site analyzed, a principal component analysis was conducted in order to reduce the dimensionality of the study. A three-component solution was chosen to best explain 70.285% of the variance (Table 6). 

The first principal component accounted for 42.838% of the variance found within the data matrix (Table 6), which was mainly caused by Cu, Cr, Ni, Co and Zn concentrations, with very high charges (0.942, 0.928, 0.927, 0.773 and 0.681, respectively), as shown in Table 7. As previously mentioned, the mean values of all elements in the sampled soil were slightly higher than the values of the natural geological background levels. This indicated that the first component collected all the information from the samples, which had heavy metal contents that originated mainly from the parent rock [53].

**Figure 2:**
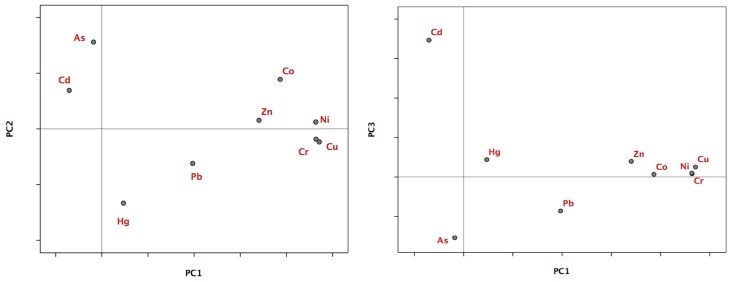
Representation of the heavy metals, in the factorial planes 1–2 (**left**) and 1–3 (**right**).

The second principal component accounted for 16.643% of the variance, with arsenic and mercury having the highest and inverse charge factors (0.780 and −0.666 respectively, Figure 2 left). The inverse sign in the changes in factors should be interpreted together with the geographical localization analysis of the samples. The general meaning is that the two metals appear in the area studied in inversely proportional concentrations. For a specific soil sample with a high concentration of As, the concentration of Hg is small in general and vice versa. As the average values of these metals were proportionally higher than the geological background levels (As = 52% and Hg = 76%), this information combined with the geographical localization analysis of the samples indicated that the second component collected information from the sites where the presence of these elements could be determined not only from the parent rock (the lithogenic factor) but also by industrial practices. 

The third principal component accounted for 10.805% of the overall information. In this, Cd had the highest load factor as it is 0.867 higher than the rest of the other metals. Within the areas sampled, the mean value of this element exceeded the natural geological background level, having a high value for the coefficient of variation (CV_Cd_ = 78%). The geographical location of the samples with the highest Cd values showed that the second component basically collected information of samples where the concentration of this element was caused by agricultural practices (and thus, being influenced by the lithological factor) (Figure 2 right).

Lead (Pb) contributed very little to the three principal components of this multivariate solution. It was known that the mean value of this element in the study area exceeded the natural geological background level and that its coefficient of variation was the highest out of all the metals studied (CV_Pb_ = 80%). This indicated that the distribution of Pb was diverse and fluctuating, with its concentration at each site potentially being conditioned by multiple factors, including human activities [54] and the parent rock. This might account for the results as within the factorial solution, Pb was the only element studied that showed low charge factors within the three principal components (0.394, −0.310 and −0.215, respectively), which determined its position within the factorial planes (Figure 2).

The Pearson correlation coefficients for the heavy metal concentrations in the soil samples (Table 8) showed a direct relationship between the majority of the metal concentrations from samples (if we exclude Cd as well as As and Hg in some cases, which had negative correlation values indicating an inverse relationship). These statistically significant levels, marked with asterisks (** *p*-value < 0.01; * *p*-value < 0.05), showed that the relationship between the metal concentrations were sometimes small but not negligible. 

Cu, Cr, Ni and Zn were the most related elements with direct connections, which indicated that these metals were generally present in the samples at the same time and in equivalent or proportional quantities, suggesting that the origin of these elements was mainly lithological. However, there was an inverse relationship between the concentrations of Cd and As with the rest of the metals. The metals, Hg and Cd, had a sporadic inverse relationship with Co. These relationships could serve to identify artificial sources caused by different human activities. 

### 3.4. Correlation between Heavy Metal Concentrations and Soil Properties

The correlation analysis between the total contents of heavy metals and some soil properties has shown (Table 9) that the clay content and the cation exchange capacity are highly correlated with the concentration of Co, Cu, Cr, Ni, Pb and Zn. This result confirmed the high affinity of specific elements with the clay minerals within the soil. However, the soil pH only showed a statistically significant relationship with the Pb concentration, while the percentage of the organic matter was found to only significantly change in proportion to the concentration of Cd.

### 3.5. Spatial Distribution of Heavy Metal Content in Soil

**Figure 3:**
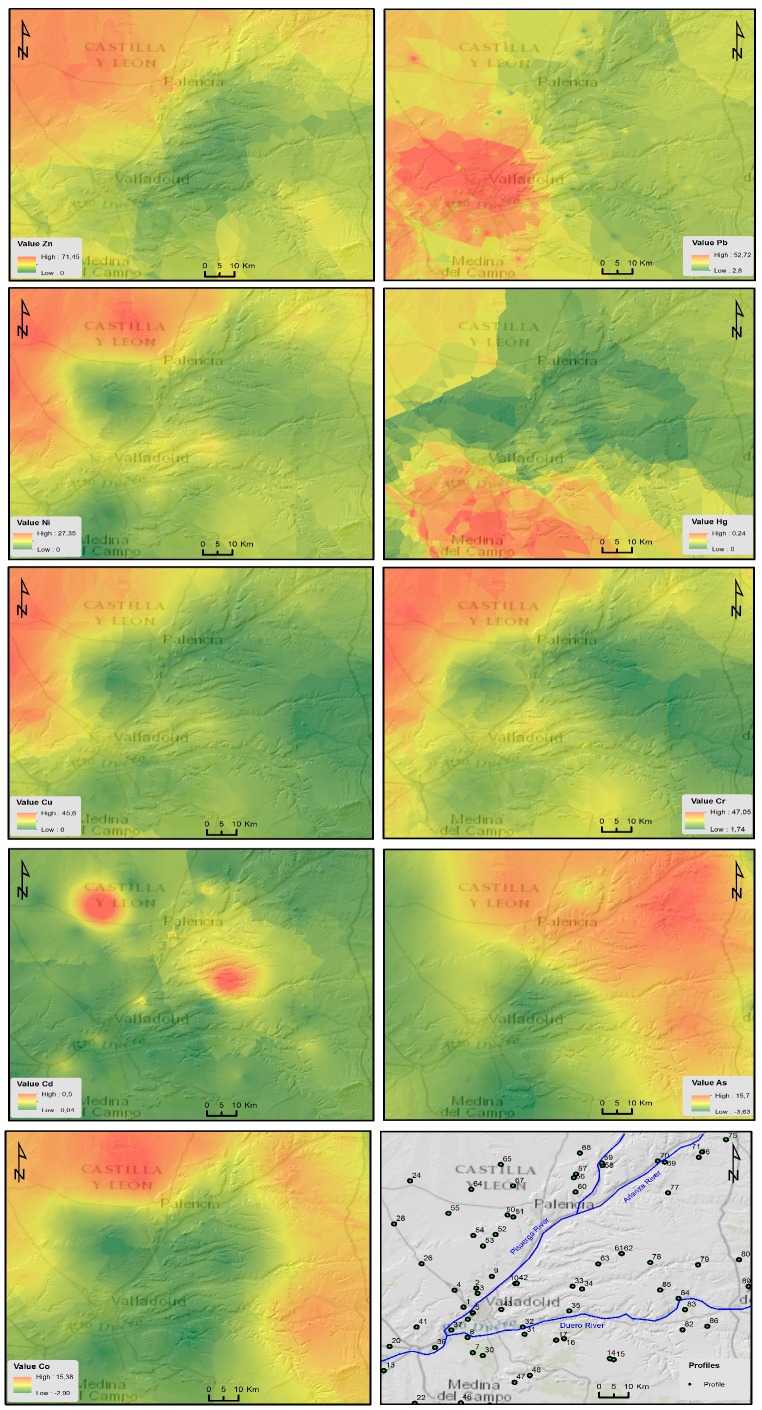
Spatial distribution of the content of the different heavy metals in the soil of the study area.

The geostatistical method of spatial interpolation (standard Kriging) allowed for the spatial distribution of the values of each metal in non-sampled sites (Figure 3) to be predicted using the values obtained from the samples collected in the field. It was shown that the closest values were more similar than those that were further apart. 

**Figure 4:**
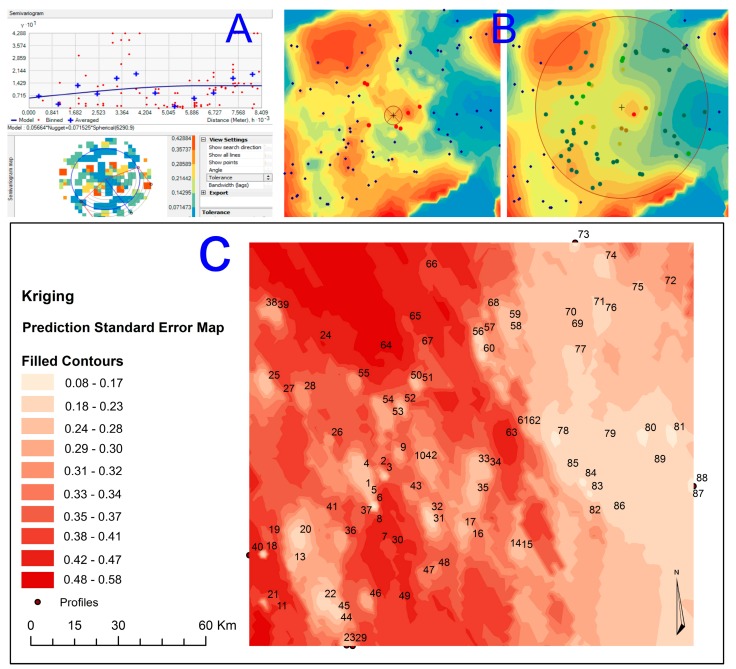
Spherical semivariogram obtained using: (**A**) exponential-type Kernel function, (**B**) Neighborhood Interpolation and (**C**) Error prediction map.

By applying the geostatistical analysis extension, we analyzed the resulting charge histogram of all the sample data and implemented the logarithmic transformation. This allowed the frequency histogram to be fitted to a normal distribution and equalized the values of the mean and median. Standard Kriging was applied with a prediction surface output and a logarithmic transformation. This process was further carried out by applying an exponential-type Kernel function and obtaining a spherical semivariogram (Figure 4A) with real anisotropy. Following this, a standard neighborhood operation (Neighborhood type) was performed with maximum neighbors of five and minimum neighbors of two samples (Figure 4B). The standard error prediction map (Figure 4C) showed maximum values between the range of 0.48–0.57 when compared with the interpolation map. 

**Figure 5:**
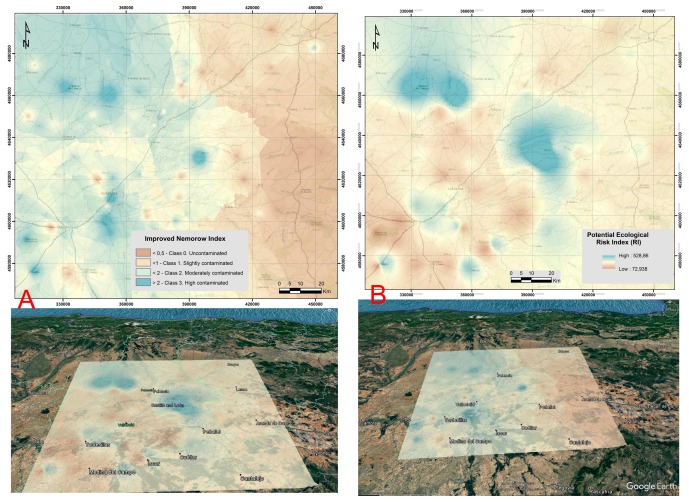
Global heavy metal pollution index measured by: (**A**) Improved Nemerow Index and (**B**) Potential Ecological Risk Index on top of the free 3D virtual globe (Google Earth).

The cartography of the risk of soil contamination from the I_NM_ (Figure 5A) showed that the highest concentrations of heavy metals were located in the north-western section of the area studied (in the city outskirts of Villalón de Campos), in soil developed on top of the sediments of the “Tierra de Campos” Facies as well as in soil situated in the center and south-eastern areas (in the surroundings of the cities of Valladolid). Uncontaminated or slightly contaminated soil was present in the eastern section of the region. 

If we consider the spatial distribution of the Potential Ecological Risk Index (RI) (Figure 5B), it can be observed that the highest levels of contamination by heavy metals were concentrated in the central area (to the east of the town of Dueñas) and north-west of the area studied (in the outskirts of Villalón de Campos), in soil developed on top of the sediments of the “Tierra de Campos” Facies as well as on Miocene limestones and loams. Other areas with moderate potential ecological risk indices were found on the city outskirts of Olmedo and Alaejos as well as between Tordesillas and Valladolid. 

## 4. Discussion

The evaluation of the environmental risk of heavy metals in the soil has been carried out through several pollution indices, a principal components analysis and the spatial distribution of nine heavy metals.

The indexes of Nemerow (IN), Pollution Factor (*Pi*), Geoaccumulation Index (Igeo), Nemerow “Improved” Index (IMN) and Individual Ecological Risk (Er) and Potential Indices (IR) have been calculated.

After taking the Nemerow index into account, the soil within the study area ranged from being contaminated to highly contaminated. However, using the “improved” Nemerow index (*I_MN_*), the soil was shown to be moderately to heavily contaminated. A large difference was observed when calculating the percentage and degree of contamination of the soil using each of the different contamination indices. In the soil of the Northern Plateau of Spain, the *I_MN_* was a better representation of the actual degree of contamination present.

The calculations based on the Potential Ecological Risk Index indicate that the majority of soil samples (96.92%) have a low to moderate ecological risk of potential contamination, while the minority (3.08%) of the soil has a considerable risk of contamination.

Therefore, both the “improved” Nemerow index and the Potential Ecological Hazard Index are the best in informing us of the degree of contamination in the surface horizons of the soil of the Northern Plateau of Spain.

**Figure 6:**
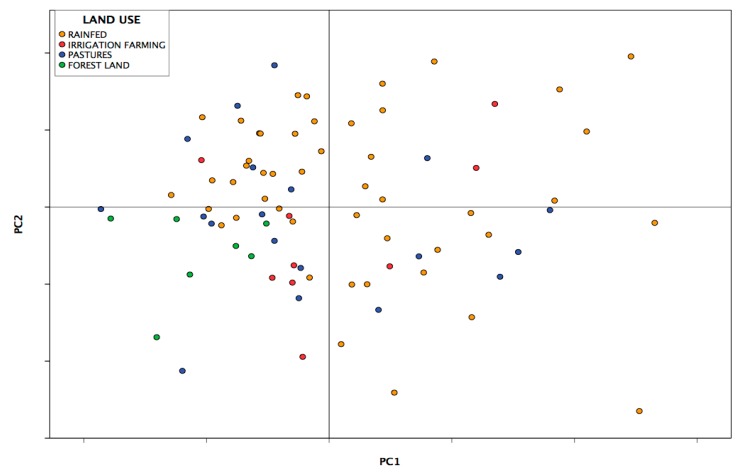
Representation of the sampling points in the factorial plane 1–2 according to land use.

The nine trace elements have been divided into three main components: CP1 (Cu, Cr, Ni, Co and Zn); CP2 (As and Hg) and CP3 (Cd). Pb has a similar and scarce contribution across all the three main components. Pearson correlation coefficients and their significance levels indicate that most concentrations of heavy metals are positively correlated with each other (with the exception of Cd correlations). Furthermore, these were all statistically significant correlations, which implies that the contents of these heavy metals have been supplied from very similar sources of contamination. The correlation coefficients of the metals belonging to the CP1 (between Cr and Cu; Ni and Cu; Cr and Ni as well as Ni and Co) indicate a strong and significant linear correlation (*p* < 0.01), providing evidence for one shared origin of these metals. The lack of significant linear correlation between Cd and other metals suggests that sources of Cd must be very different from those of other metals. These data suggest that concentrations of heavy metals could be affected by more than one source of pollution, including the following: from the parent rock itself, from agricultural activities and from the discharge of wastewater into rivers and streams. It is hypothesized that the contamination is caused by industrial development and increasing population density (Figure 6).

A number of soil parameters, such as organic matter content, pH, clay content and cation exchange capacity, have been measured to determine their influence on or correlation with the content of heavy metals. By conducting the correlation analysis, the clay content and cation exchange capacity (CEC) were found to be significantly correlated with Co, Cu, Cr, Ni, Pb and Zn concentrations. This confirms the high affinity of certain elements with the minerals of the soil clay. However, soil pH is significantly correlated only with Pb concentration. It was found that the concentration of the heavy metals increases in the more clay-like horizons of the soil (Bt horizons of the Alfisols and Bw of the Inceptisols), which can easily be explained if we take into account that the adsorption capacity of these elements in soil depends on a number of properties, such as clay content, organic matter and cation exchange capacity.

A mapping, of the spatial distributions of heavy metal contents in the soil of the Northern Plateau (Spain) has been carried out using by interpolation methods (Kriging).

The distribution of the heavy metals (Ni, Cu, Cr, and Zn) was very similar and all had high concentrations in the soil. These heavy metals were found in the north-western section of the study area in soil that had developed on top of the sediments of the “Tierra de Campos” facies. High values for these elements were also observed in the soil overlaying Miocene limestones as well as on top of the terraces, alluvial deposits of the rivers and streams that run through the area. The concentrations of these metals were influenced by the contents of the parent rock. Furthermore, the concentrations of Cu and Zn could have been influenced by agricultural practices (fertilized with waste, fertilizers and chemical pesticides). The highest Pb content was found within the surroundings of Valladolid, the most populated and industrial city [55], in soil on top of terraces as well as alluvial deposits of rivers and on top of the clay-like sandstones of the “Tierra de Campos” Facies. Lead in the soil came mainly from vehicle exhaust emissions, industrial sources and the run-off of residual waters into rivers and streams. The content of Hg was dispersed throughout the entire study area with most of the content found in the south-western section of the region on top of the terraces of the River Duero. This element might have a natural (parent rock) and anthropogenic origin (atmospheric residues coming from the incineration of waste products). The distribution of Co decreased in a concentric manner from the periphery towards the south-eastern section of the study area, with the highest amount of content located in the developed soil overlaying sediments of the “Tierra de Campos” Facies (provinces of Palencia and Burgos). Co in the soil came mainly from the parent rock. With regards to Cd, its content was distributed as two concentric circles, with one situated toward the north-eastern part of Valladolid in soil that had developed on top of limestone wastelands and chalky loams (Cuestas Facies). The other circle contained the highest concentration of Cd in the region, which was measured in the soil taken from an area surrounding the electric transformer–accumulator of Fuentes de Nava (Palencia). It is worth noting that Cd is used in nickel–cadmium accumulators. The highest concentrations of As were situated in the north-eastern corner of the study area in soil that had developed over the terraces and alluvial deposits of the rivers Pisuerga, Arlanza, Arlanzón, Carrión, and Riaza in the provinces of Palencia and Burgos. The origin of As may have been lithogenic as well as also being due to the application of manure, fertilizers and pesticides. 

The overall analysis of the soil contamination in the study area was carried out using two contamination indices: the improved Nemerow index (I_NM_) and the potential ecological risk index (RI). Both reflect the general situation of the contamination caused by the simultaneous presence of the nine heavy metals. This was useful for evaluating the possible sources of the heavy metals in the area studied and for identifying the soil that contained the highest concentrations of the metals. 

## 5. Conclusions

The correlation analysis between the total contents of heavy metals and some soil properties has shown that the clay content and the cation exchange capacity are highly correlated with the concentrations of Co, Cu, Cr, Ni, Pb and Zn.

According to the multivariate statistics, the metals from the soil of the region studied are related with three principal components. Of these, the first one is closely related to Cu, Cr, Ni, Co and Zn. The second principal component has a strong relationship with As and Hg, while the third is explained primarily by Cd. According to its origin and dominant substrates, lead has a similar contribution and is scarcely represented within the three main components in the multivariate solution.

On the basis of the Pearson correlation coefficients and the levels of significance for the contents of heavy metals in the soil samples, it can be deduced that most of the levels are positively correlated with each other (except with the correlations of Cd). In addition, the correlations are statistically significant, which implies that the contents of these metals have originated from similar sources of pollution and could be affected by more than one source of contamination.

Taking into account the values obtained from heavy metals, analysis of main components and their spatial distribution, it can be concluded that a determinant of concentrations of most metals in the soil of the Northern Plateau of Spain is the nature of the mother rock. Some metals, such as Cu, Zn and As, have also increased their content in soil due to certain agricultural activities (fertilizers and chemical pesticides). Hg is related to soil that has developed on alluvial deposits and irrigated soil. The soil that has developed on limestones and calcareous sediments has a high Cd content and the greater concentration (anomaly) of Cd in the region occurs in the vicinity of an electric transformer of an urban nucleus. The highest concentration of Pb occurs in the vicinity of Valladolid due to the gases from the vehicles, industrial sources as well as the discharge of waste water to rivers and streams. In accordance with the improved Nemerow index, most of the soil of the Northern Plateau of Spain are considered to be between “between non-contaminated to moderately contaminated”. According to the Potential Ecological Risk Index, the soil presents a general level of risk that varies from “low to moderate”. 

The results of this study demonstrate that agricultural production can continue, but there should be some improvement measures to protect the security of agricultural products. This might include a reduction in the amount of fertilizers and pesticides used as well as the application of treatments to the soil in some places. In addition, the results obtained in this research should be useful for establishing future land planning guidelines or for providing recommendations for land use within this region. 

## Figures and Tables

**Table 1:** Statistical summary of soil orders: mean ($\bar{x}$) and range (R).

Soil Order		% Clay	pH-H_2_O	% Organic Matter	CEC cmol/kg
**Entisols**	$\bar{x}$	27.8	7.45	1.3	14.2
	R	0.6–45.9	7.0–7.7	0.1–2.98	0.6–31.0
**Inceptisols**	$\bar{x}$	21.4	7.01	0.6	14.2
	R	4.1–41.0	5.8–8.2	0.07–1.41	1.9–31.4
**Alfisols**	$\bar{x}$	27.1	7.1	0.96	16.0
	R	1.5–62.1	4.9–8.1	0.16–2.55	0.7–37.5

**Table 2:** Statistical analysis of heavy metals in soil of the Northern Plateau of Spain.

	As	Cd	Co	Cu	Cr	Hg	Ni	Pb	Zn
Mean ($\bar{x}$)	6.71	0.08	6.49	17.19	18.68	0.08	12.05	14.10	35.31
Geometric mean (GM)	5.78	0.07	5.67	14.00	14.75	0.07	10.24	10.92	31.65
Median (Me)	6.50	0.06	6.15	14.55	15.60	0.07	11.01	9.96	34.50
Minimum (min)	1.80	0.04	1.30	1.00	1.74	0.01	2.11	2.25	4.16
Maximum (max)	15.70	0.38	17.84	48.90	51.50	0.45	37.20	73.04	71.45
Standard deviation (S)	3.51	0.06	3.21	10.69	12.28	0.06	6.76	11.32	14.63
Coefficient of variation (CV)	0.52	0.69	0.49	0.62	0.66	0.76	0.56	0.80	0.41
Kurtosis	−0.31	11.27	0.23	0.86	−0.15	18.30	1.48	6.75	−0.52
Natural geological Background	5.67	0.07	4.18	11.20	12.53	0.05	8.63	9.39	21.17
World mean	20	0.40	8	12	50	0.10	25	15	40
World ranks	0.1–50	0.01–2	0.5–0.65	1–200	2–1500	0.01–0.5	2–500	2–200	1–800

**Table 3:** Pollution factor and Nemerow index of the heavy metals.

	*Pi* = C/B As	*Pi* = C/B Cd	*Pi* = C/B Co	*Pi* = C/B Cu	*Pi* = C/B Cr	*Pi* = C/B Hg	*Pi* = C/B Ni	*Pi* = C/B Pb	*Pi* = C/B Zn	*Pi_max_*	*Pi_ave_*	I_N_
Mean ($\bar{x}$)	1.18	1.16	1.55	1.54	1.49	1.54	1.40	1.50	1.67	2.75	1.45	2.20
Median (Me)	1.15	0.86	1.47	1.30	1.25	1.30	1.28	1.06	1.63	2.52	1.35	2.06
Minimum (min)	0.32	0.57	0.31	0.09	0.14	0.19	0.24	0.24	0.20	0.78	0.47	0.65
Maximum (max)	2.77	5.43	4.27	4.37	4.11	8.41	4.31	7.78	3.38	8.41	2.98	6.26
Standard deviation (S)	0.62	0.80	0.77	0.95	0.98	1.17	0.78	1.21	0.69	1.31	0.49	0.96

**Table 4:** Geo-accumulation index (I_geo_) of the heavy metals.

	Igeo									Igeo_ave_	Igeo_max_	I_NM_
	As	Cd	Co	Cu	Cr	Hg	Ni	Pb	Zn			
Mean ($\bar{x}$)	−0.50	−0.34	−0.02	−0.30	−0.31	0.16	−0.23	−0.05	−0.05	0.18	0.91	0.79
Median (Me)	−0.33	−0.58	0.10	−0.24	−0.23	0.15	−0.13	−0.19	0.07	−0.18	0.90	0.72
Minimum (min)	−2.18	−1.17	−2.14	−4.10	−3.39	−2.66	−2.51	−2.33	−2.98	1.82	−0.85	0.26
Maximum (max)	0.94	2.08	1.64	1.51	1.50	2.85	1.63	2.69	1.12	0.77	2.85	2.07
Standard deviation (S)	0.82	0.67	0.79	0.99	1.05	0.81	0.87	1.02	0.75	0.49	0.65	0.37

**Table 5:** Individual (*Er*) and potential (*RI*) Ecological Risk Index of the soil studied.

	*Er* As	*Er* Cd	*Er* Co	*Er* Cu	*Er* Cr	*Er* Hg	*Er* Ni	*Er* Pb	*Er* Zn	*RI*
Mean ($\bar{x}$)	11.84	34.65	7.76	7.62	2.98	61.77	6.88	7.51	1.64	140.30
Median	11.46	25.71	7.36	6.49	2.49	51.85	6.31	5.30	1.62	134.45
Minimum (min)	3.17	17.14	1.56	0.45	0.28	7.41	1.22	1.20	0.20	3.17
Maximum (max)	27.69	162.86	21.34	21.83	8.22	336.30	21.55	38.89	3.38	486.70
Standard deviation (S)	6.18	24.08	3.83	4.80	1.96	46.79	3.98	6.03	0.72	57.83

**Table 6:** Cumulative variance and variance of the major components.

Component	Initial Eigenvalues		
	Total	% Variance	% Accumulated
**1**	3.855	42.838	42.838
**2**	1.498	16.643	59.481
**3**	0.972	10.805	70.285
**4**	0.858	9.533	79.818
**5**	0.789	8.762	88.580
**6**	0.596	6.623	95.204
**7**	0.265	2.945	98.149
**8**	0.098	1.087	99.236
**9**	0.069	0.764	100.000

**Table 7:** Matrix for the principal component analysis of heavy metals.

Component	Components		
	1	2	3
Copper	0.942	−0.116	0.063
Chrome	0.928	−0.092	0.021
Nickel	0.927	0.062	0.025
Cobalt	0.773	0.445	0.017
Zinc	0.681	0.078	0.099
Lead	0.394	−0.310	−0.215
Arsenic	−0.036	0.780	−0.385
Mercury	0.094	−0.666	0.110
Cadmium	−0.141	0.346	0.867

**Table 8:** Pearson correlation matrix for the heavy metal concentrations in the soil samples.

	*As*	*Cd*	*Co*	*Cu*	*Cr*	*Hg*	*Ni*	*Pb*	*Zn*
***As***	1	0.100	0.260 *	−0.235 *	−0.145	−0.252 *	−0.075	−0.148	−0.020
***Cd***	0.100	1	−0.040	−0.182	−0.178	−0.111	−0.156	−0.117	−0.048
***Co***	0.260 *	−0.040	1	0.629 **	0.620 **	−0.050	0.758 **	0.129	0.504 **
***Cu***	−0.235 *	−0.182	0.629 **	1	0.897 **	0.080	0.894 **	0.364 **	0.586 **
***Cr***	−0.145	−0.178	0.620 **	0.897 **	1	0.088	0.863 **	0.381 **	0.559 **
***Hg***	−0.252 *	−0.111	−0.050	0.080	0.088	1	0.010	0.094	0.055
***Ni***	−0.075	−0.156	0.758 **	0.894 **	0.863 **	0.010	1	0.300 **	0.478 **
***Pb***	−0.148	−0.117	0.129	0.364 **	0.381 **	0.094	0.300 **	1	0.196
***Zn***	−0.020	−0.048	0.504 **	0.586 **	0.559 **	0.055	0.478 **	0.196	1

**, *: level of significance *p* < 0.01 and *p* < 0.001, respectively.

**Table 9:** Coefficient of correlation of the concentrations of trace elements with the properties of the soils (**, ***: level of significance being *p* < 0.01 and *p* < 0.001, respectively).

Element	pH	% Organic Matter	% Clay	CEC
**As**	0.23	0.12	0.01	0.09
**Cd**	0.25	0.59 ***	0.18	0.08
**Co**	0.23	0.04	0.63 ***	0.79 ***
**Cu**	0.30	0.06	0.76 ***	0.88 ***
**Cr**	0.32	0.12	0.84 ***	0.93 ***
**Hg**	0.17	0.12	0.05	0.1
**Ni**	0.26	0.01	0.72 ***	0.85 ***
**Pb**	0.51 **	0.12	0.50 **	0.52 **
**Zn**	0.32	0.16	0.77 ***	0.89 ***

## Supplementary Materials

The following are available online at www.mdpi.com/1660-4601/14/6/568/s1, Nemerow Index Kmz, RI Index Kmz and HTML File-Stadistical Kriging.
